# Clinical management of alveolar osteitis. A systematic review

**DOI:** 10.4317/medoral.24256

**Published:** 2021-10-27

**Authors:** Federico Garola, Gerardo Gilligan, René Panico, Nicolás Leonardi, Eduardo Piemonte

**Affiliations:** 1 DDS, Oral Medicine Department, Facultad de Odontología, Universidad Nacional de Córdoba, Argentina; 2 DDS, PhD, Assistant Professor, Oral Medicine Department, Facultad de Odontología, Universidad Nacional de Córdoba, Argentina; 3 DDS, PhD, Head Professor, Oral Medicine Department, Facultad de Odontología, Universidad Nacional de Córdoba, Argentina; 4 DDS, Oral Medicine Department, Escuela de Odontología, Facultad de Ciencias de la Salud, Universidad Católica de Córdoba, Argentina; 5 DDS, PhD, Associate Professor, Oral Medicine Department, Facultad de Odontología, Universidad Nacional de Córdoba, Argentina

## Abstract

**Background:**

Alveolar Osteitis (AO) is one of the most common complications of tooth extraction. Several therapeutic interventions have been described for the treatment of AO, however, there are no treatment standardized protocols. The aim of this study was to conduct a systematic review on the efficacy in pain control of the different treatments for AO. The feasibility of the application of these interventions is also discussed.

**Material and Methods:**

A structured electronic and hand search strategy was applied to PubMed, Scopus, Cochrane Library, OpenGrey, and Google Scholar between January 2010 and July 2020 to identify studies according to PRISMA guidelines. The inclusion criteria were original English and Spanish clinical trials that analyzed pain-control parameters according to visual analog scale (VAS, 0-10 scale), or pain relief patients’ percentages. Those treatments that reach VAS ≤ 4 on day 2 or before; or ≥ 85% of patients with absence of pain symptoms at day 7 or before were considered accepTable for their recommendation.

**Results:**

The final review included 17 clinical trials. Among them, there were analyzed a total of 39 different AO treatments. 53,8% of the treatments fulfill the proposed parameters for pain control.

**Conclusions:**

Treatment alternatives are multiple, heterogeneous, and difficult to compare. The management of AO is summarized in basic (intra-alveolar irrigation) and specific procedures (Alveogyl®, Neocones®, SaliCept Patch®, Low-Level Laser, Platelet-Rich Fibrin) that reach pain control success. They could be selected according to their availability and advantages or disadvantages.

** Key words:**Dry socket, alveolar osteitis, treatment, management, pain control, pain relief.

## Introduction

Alveolar osteitis (AO), localized osteitis, or usually so-called dry socket, is one of the most common complications of tooth extraction (1), with a frequency of 1 to 5% (2). In 30% of the cases, AO is frequently associated with extractions of mandibular third molars (3). AO is defined by the presence of postoperative pain in and around the post-extraction site, which increases in intensity between 1 and 3 days after extraction, accompanied by a partially or totally disintegrated blood clot within the alveolar socket, with or without evident halitosis (4). Regarding AO etiopathogenesis, it was described as partial or total fibrinolysis, possibly triggered by direct (physiological) or indirect (non-physiological) activating substances. After surgical trauma, alveolar bone cells release direct activators, while indirect activators are secreted by bacteria (5). Consequently, a necrotic socket in the absence of blood vessels and granulation tissue could alter alveolar healing.

Risk factors for AO are associated with difficult or traumatic extractions, female gender, tobacco use, oral contraceptive use, and pre-existing infection at the extraction site. The incidence of AO could be reduced by controlling these factors. Local application of chlorhexidine could be also useful (6).

Several therapeutic interventions have been described for the treatment of AO. In the 60s, the first reports of AO described the placement of zinc oxide (7). New therapeutic approaches were developed during the last decades. Among them, platelet-rich fibrin (PRF) is currently widely used in AO cases (8,9). However, there are no treatment standardized protocols. The aim of this study was to perform a systematic review of the AO treatment considering pain control parameters and feasibility of application. The analysis of these data could be the first step to design a clinical guide for the management of AO by general dentists.

Materials and Methods

A systematic review was performed in accordance with PRISMA declaration (10) (Preferred Reporting Items for Systematic Review and Meta-Analysis) to gather available and current evidence of AO treatment.

- Search strategy

Comprehensive electronic searches between January 2010 and July 2020 were performed. The searches were conducted in the following electronic databases: Pubmed-Medline, Scopus, and Cochrane Library. For searching the grey literature, OpenGrey and Google Scholar were also assessed. In addition, hand searches of relevant journals, such as those listed in other systematic reviews, were performed.

The search strategies for each database were as follow: In Cochrane Library, the search was performed using the following keywords: (dry socket treatment) or (dry socket management) or (alveolar osteitis treatment) or (alveolar osteitis management) or (treatment of dry socket) [all fields] [content type: trials]. In PubMed, the search was performed using the following keywords: (dry socket treatment) or (dry socket management) or (alveolar osteitis treatment) or (alveolar osteitis management) or (treatment of dry socket) and (trial) [title/abstract]. In Scopus, the search was performed using the following keywords: (dry socket treatment) or (dry socket management) or (alveolar osteitis treatment) or (alveolar osteitis management) or (treatment of dry socket) (limit-to (exactkeyword, “human”)) or (limit-to (exactkeyword, “humans”)) [all fields]. In Google Scholar, the search was performed using the following keywords: (management of dry socket) (without the words (preventive)) [in the title of the article]. In Open Grey, the search was performed using the following keywords: (dry socket treatment) OR (dry socket management) or (alveolar osteitis treatment) or (alveolar osteitis management) or (treatment of dry socket).

- Eligibility criteria

The present review focused on the following research question: What is the optimal clinical management for alveolar osteitis? PICOS (Population, Intervention, Comparison, Outcome, and Studies) schema for all the included studies to elaborate upon this research question were used to establish the eligibility criteria as follows:

Population: Adults patients with diagnosis of AO.

Intervention: Treatment or management of AO by intra-alveolar clinical procedures.

Comparison: Other treatment or management of AO by intra-alveolar clinical procedures or absence of procedures.

Outcome: Pain level after treatment of AO by intra-alveolar clinical procedures.

Studies: Randomized and non-randomized clinical trials.

- Inclusion criteria

The search strategy was restricted to original English and Spanish languages. Inclusion criteria were clinical trials that analyzed pain-control parameters according to visual analog scale (VAS, 0-10 scale) or pain relief patients’ percentages.

- Exclusion criteria

Preventive treatments, AO treated solely by antibiotics, analgesic-opiates or oral mouthwashes administration, deficient clinical data, lack of data regarding pain control, or records whose categorization was not adapted to the inclusion criteria were not included after full-text reading.

- Data extraction

Two independent researchers (F.G and G.G) conducted data extraction and validity assessment of the studies that met the inclusion criteria. Any discrepancy between the researchers was discussed with a third researcher (E.P) until consensus was reached. Relevant information for each study was entered into a predesigned data extraction form.

- Risk of bias in individual studies

The risk of bias of the included randomized controlled trials (RCTs) was assessed by the Cochrane Risk of Bias (RoB) tool (11). “High”, “low,” or “unclear” risk scores were based on the randomization method, allocation concealment, blinding of participants, personnel, and outcome assessors, completeness of outcome data, selective reporting, and other bias. Then, the overall risk of bias for each study was reported using the following criteria:

Low risk of bias: all domains are judged to be at low risk of bias.

Unclear risk of bias: one or more domains judged to be at unclear risk of bias.

High risk of bias: one or more domains judged to be at high risk of bias.

The risk of bias in non-randomized studies of interventions (ROBINS-I) (12) was used to assess the non-RCTs included. This tool evaluates the following domains: bias due to confounding, bias in selection of participants into the study, bias in classification of interventions, bias due to deviations from intended interventions, bias due to missing data, bias in measurement of outcomes, and bias in selection of the reported result. Finally, the overall risk of bias for each study was reported using the following criteria:

Low risk of bias: if the study was at low risk of bias for all domains.

Moderate risk of bias: if the study was at low or moderate risk of bias for all domains.

Serious risk of bias: if the study was at serious risk of bias in at least one domain, but not at critical risk of bias in any domain.

Critical risk of bias: if the study was at critical risk of bias in at least one domain.

No information on which to base a judgment about risk of bias: if there was a lack of information in one or more key domains of bias.

The reviewers compared evaluations, resolved disagreements by consensus, and reported their assessments using Review Manager software (RevMan 5.4, The Nordic Cochrane Centre, Copenhagen, Denmark) for RCTs. Robvis tool (visualization tool for risk of bias assessments in a systematic review) was used for presenting the non-RCTs data as appropriate.

- Type of treatments

Treatments were classified as invasive or non-invasive due to the different nature and heterogenicity of the included studies. Invasive treatments were considered when the treatment procedure included bone curettage or suture, while the non-invasive ones did not perform these measures, whether or not they needed local anesthesia. Subsequently, treatments were also categorized as high and low complexity. High complexity treatment was considered when specific equipment and training were required (such as laser or PRF).

- Summary measures

Those treatments which showed pain reduction according to VAS of at least 4 (on a scale of 0 to 10, with 10 being the highest pain score) in the 48 hours after the first session, were considered recommended. Likewise, those treatments that reach the average percentage of patients with absence of pain symptoms during a week were also considered accepTable for this review.

A meta-analysis was not performed due to heterogeneity of the results and the lack of measures of dispersion.

## Results

**Figure 1:**
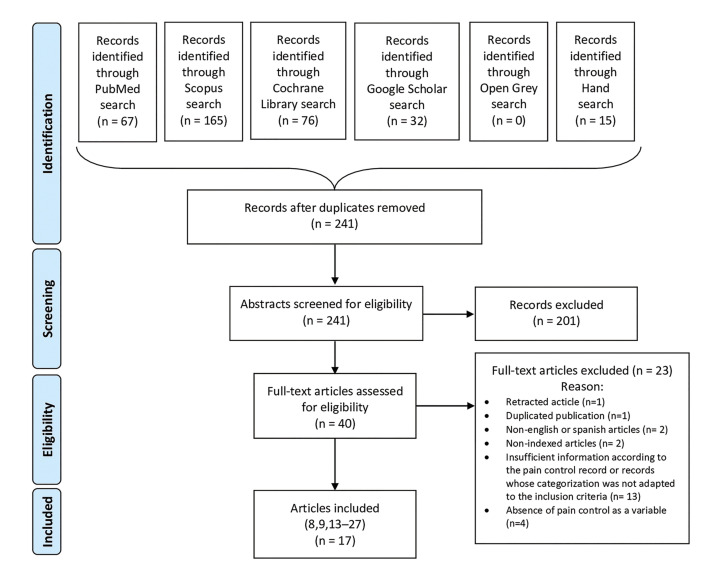
PRISMA flowchart of systematic review search process.

In the initial database search, 355 records were identified, out of which 114 were eliminated because were duplicates. After the first screening, 201 records were excluded because they had no direct relationship with the subject or they were not clinical trials. Thus, 40 records were eligible for full-text reading; of these, 1 was a retracted article, 1 was removed for double publication, 2 were not in English or Spanish language, 2 were non-indexed articles, 4 did not register pain control as a variable as well as 13 others studies had lack of data regarding pain control or records whose categorization was not adapted to the inclusion criteria. Finally, there were included 17 studies (8,9,13-27). Fig. 1 shows the flowchart of the systematic review search process. Among them, there were analyzed 1138 patients with AO and 39 different treatment protocols.

Table 1 summarizes the included studies in this review.

- Type of treatments

56.4% of AO treatments were considered as non-invasive. Among them, 81,8% were classified as low complexity ones, while 18,2% were classified as high complexity. The remaining 43.6% of AO treatments were considered as invasive. Among them, 64,7% were classified as low complexity ones, while 35,3% were classified as high complexity.

- Risk of bias across studies

**Figure 2:**
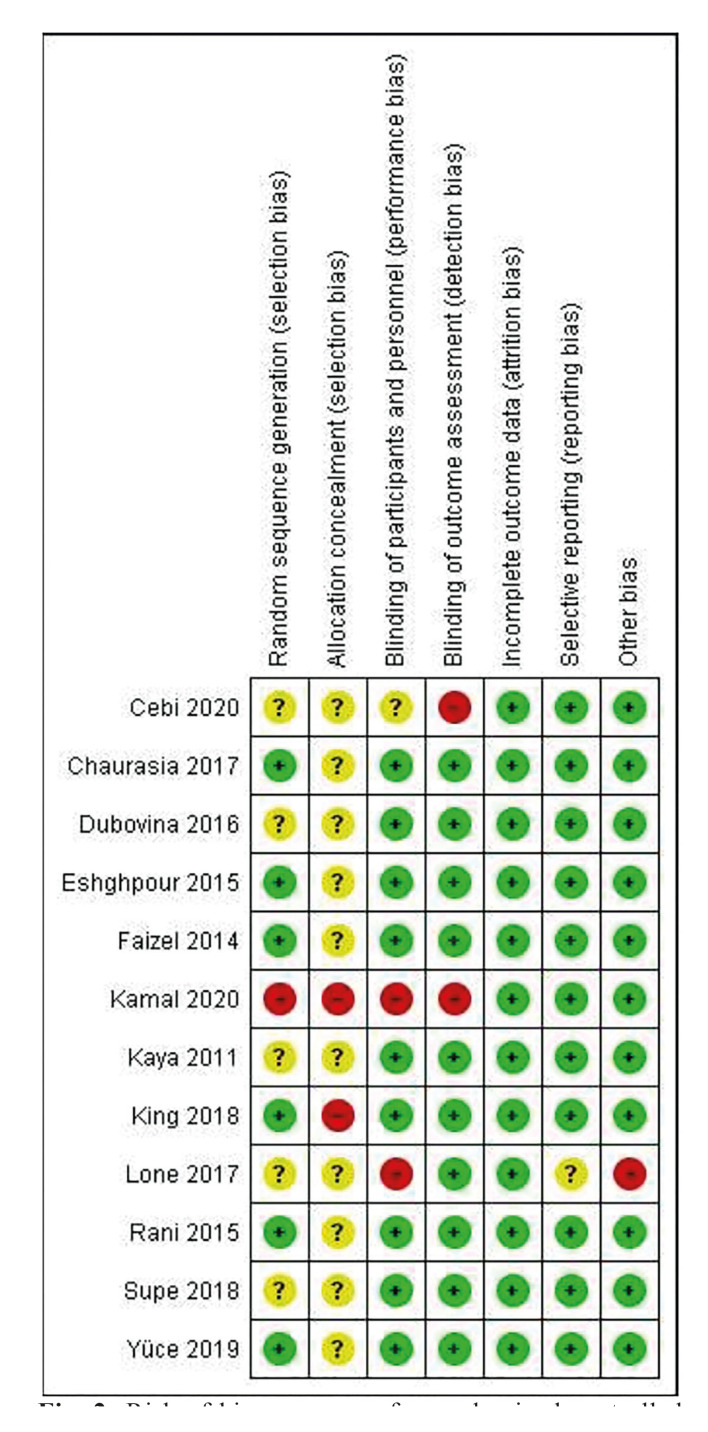
Risk of bias summary for randomized controlled trials, assessed using the Cochrane Risk of Bias (RoB) tool: review authors' judgments about each risk of bias item for each included study (+ = low; − = high;? = unclear).

Among the 12 RCTs included studies (8,13,15-19,22-25,27), 8 studies (15,17,18,22-25,27) were classified as unclear and 4 studies (8,13,16,19) were classified as high risk. The most frequent domain causing downgrading was allocation concealment. The risk of bias summary for RCTs is shown in Fig. 2.

**Figure 3:**
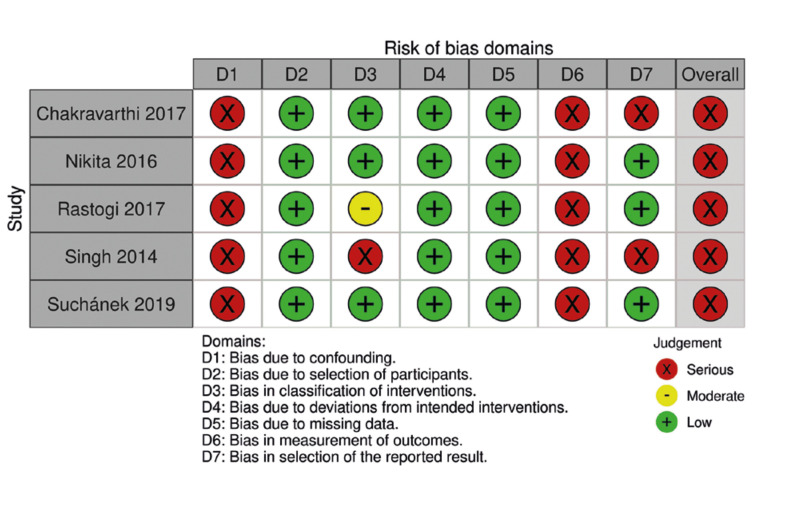
Risk of bias summary for non-randomized controlled trials, assessed using The Risk Of Bias In Non-randomized Studies of Interventions ROBINS-I tool.

The 5 non-RCTs included studies (9,14,20,21,26) were classified as serious risk. The domains that most frequently caused downgrading were bias due to confounding and bias in the measurement of outcomes. The risk of bias summary for non-RCTs is shown in Fig. 3.

**Table 1:**
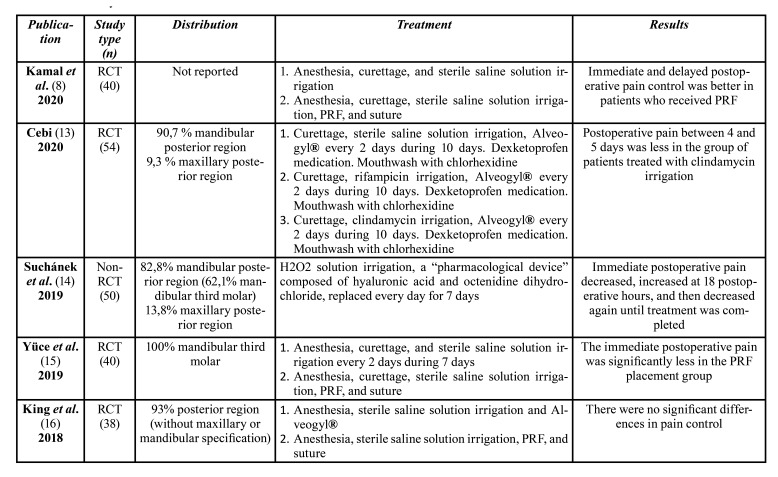
Summary of included randomized and non-randomized clinical trials.

**Table 1 cont.**
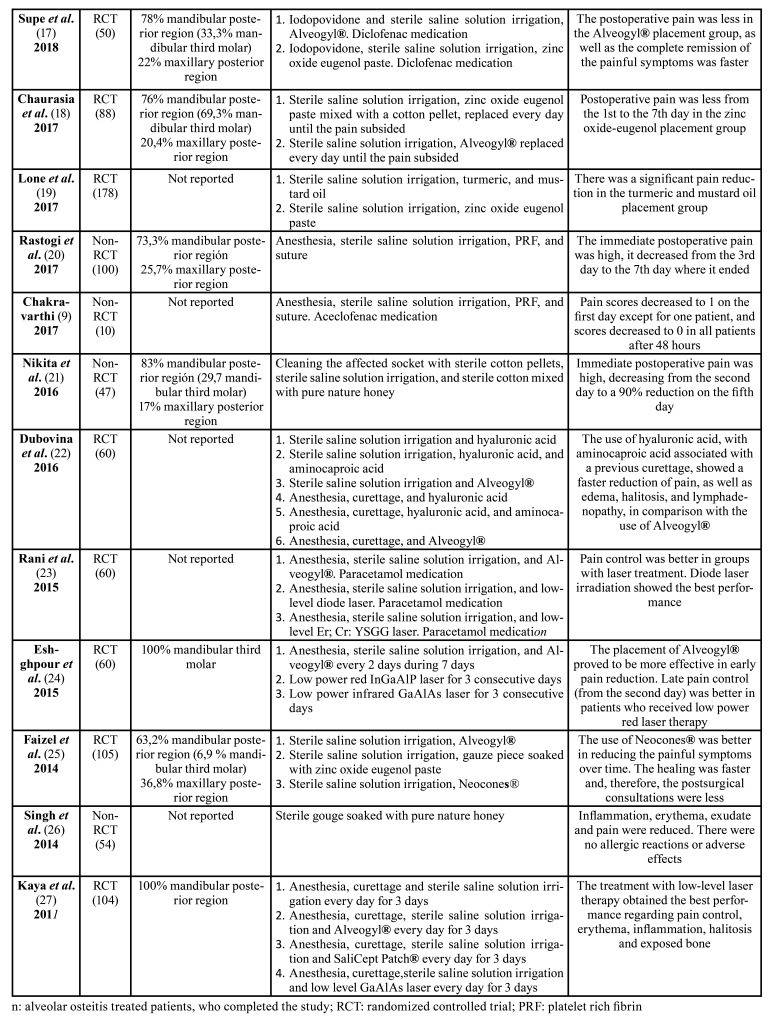
Summary of included randomized and non-randomized clinical trials.

- Pain control

Table 2 shows pain control registered using VAS and pain relief patient’s percentage registered in a 7-8-day period from the first consultation (day 0). The average of the percentages of patients without pain at one week of each treatment was 85%. This value was considered as a cut-off point of recommendation. 53,8% of the treatments fulfill the proposed parameters for pain control.

- First visit clinical procedures and total sessions required

Table 3 shows the characteristics of alveolar osteitis treatments, comparing the number of clinical procedures performed at the first visit, the number of total sessions required during a week (suture removal was considered as a session, check-up visits without clinical interventions were not considered as a session), and administrated medication.

58.9% of the treatments required at least three clinical procedures in the first visit and 69,2% of the treatments required at least three total treatment sessions.

- Pain control and total number of sessions

Comparing Tables that evaluate pain control with the treatments that require fewer total sessions, the results (best performance treatments) are described below:

1) Low complexity non-invasive treatments, with fewer sessions required, that fulfill the proposed parameters for pain reduction:

Sterile saline solution irrigation and placement of Alveogyl®. Three treatment sessions required (25).

Sterile saline solution irrigation and placement of Neocones®. Two treatment sessions required (25). This is the treatment that achieved the best pain control with fewer sessions required.

2) High complexity non-invasive treatments, with fewer sessions required, that fulfill the proposed parameters for pain reduction:

Anesthesia, sterile saline solution irrigation, and low-level diode laser irradiation. Anti-inflammatory analgesic medication with paracetamol. One treatment session required (23).

3) Low complexity invasive treatments, with fewer sessions required, that fulfill the proposed parameters for pain reduction:

Anesthesia, curettage, and sterile saline solution irrigation. Placement of SaliCept Patch®. Three treatment sessions required (27).

4) High complexity invasive treatments, with fewer sessions required, that fulfill the proposed parameters for pain reduction:

Anesthesia, curettage, and irrigation with sterile saline solution. Placement of PRF and suture. Two treatment sessions required (8).

Anesthesia, irrigation with sterile saline solution, placement of PRF, and suture. Two treatment sessions required (20).

**Table 2:**
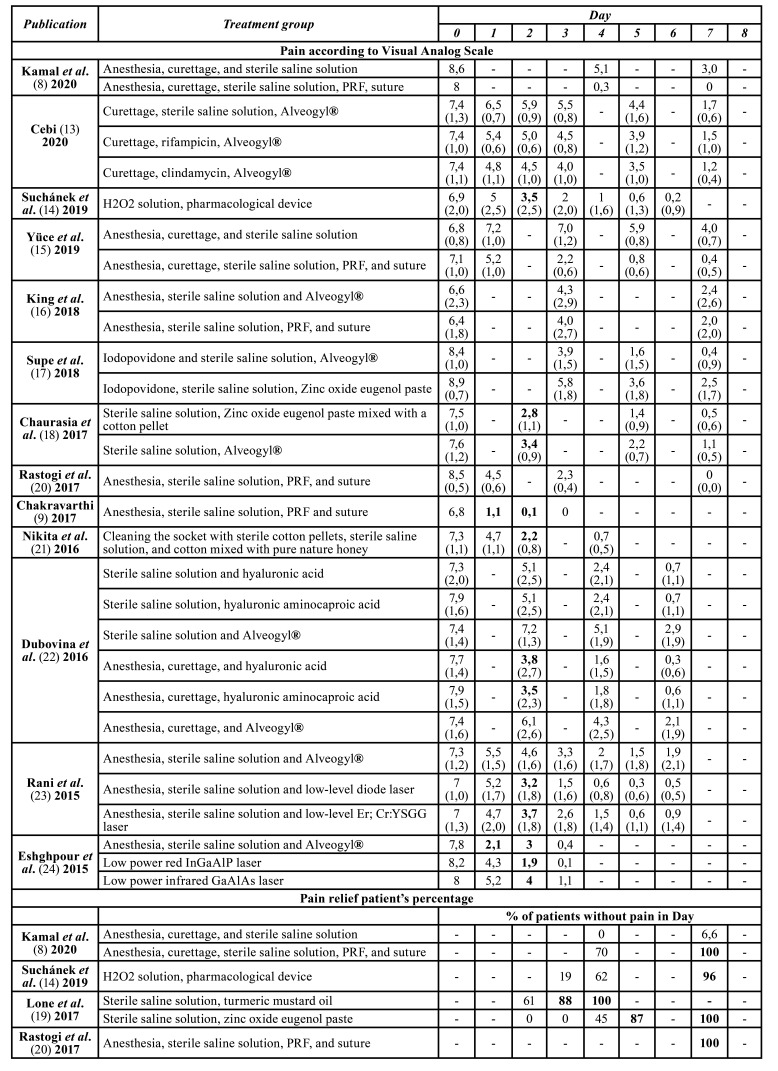
Pain control of the treatments according to Visual Analog Scale and pain relief patients’ percentages.

**Table 2 cont.**
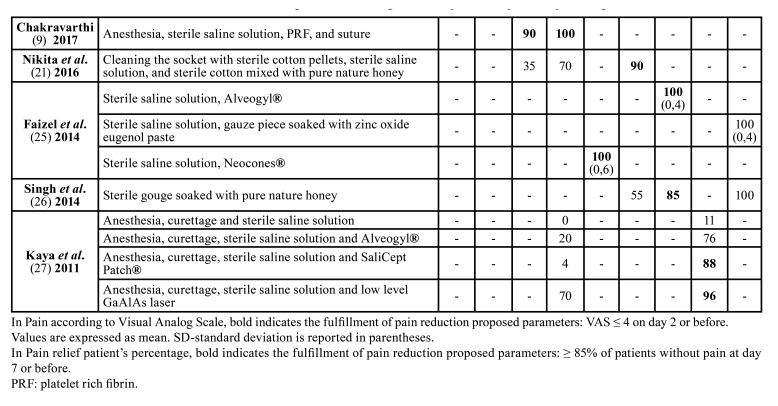
Pain control of the treatments according to Visual Analog Scale and pain relief patients’ percentages.

**Table 3:**
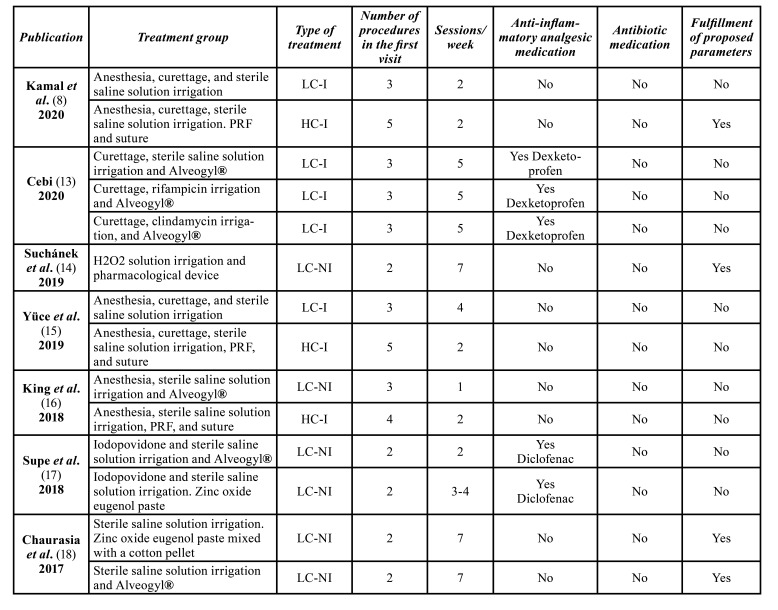
Characteristics of alveolar osteitis treatments and fulfillment of pain reduction proposed parameters.

**Table 3 cont.**
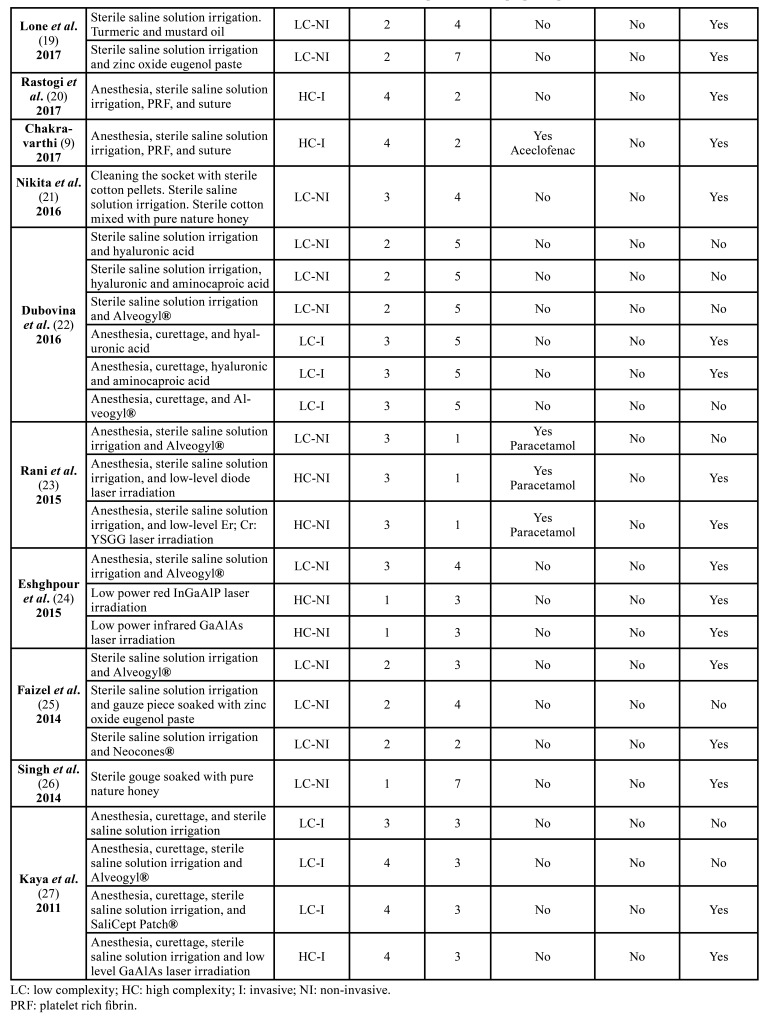
Characteristics of alveolar osteitis treatments and fulfillment of pain reduction proposed parameters.

Sterile saline solution irrigation. Placement of PRF and suture. Anti-inflammatory analgesic medication with aceclofenac. Two treatment sessions required (9). This is the treatment that achieved the best pain control with fewer sessions required.

## Discussion

Most of the current studies which address AO focus on the prevention and incidence reduction of this condition. Nonetheless, no consensus protocols on the treatment of AO were found, and choosing the best therapeutic option is still challenging for clinicians.

During the research and analysis of the consulted literature for this systematic review, a wide range of available treatments and variables were evident when evaluating therapeutic success. Consequently, we found several difficulties when comparing treatments. Pain is considered the most important symptom of AO which can vary in frequency and intensity leading the professional consultation (17). Thus, pain reduction was one of the clinical parameters considered for the analysis of these articles. An ideal AO treatment should get a faster remission of the intensity and duration of pain.

Regarding AO treatments analyzed in this study, those that showed the best performance used intra-alveolar irrigation (with sterile saline solution or iodopovidone) prior to other therapeutic procedures. Intra-alveolar irrigation offers many advantages such as microbial load reduction and necrotic tissue or clot debris removal. Almost all of the included studies used intra-alveolar irrigation as an early measure, complemented by other therapeutic approaches of different complexity. Interestingly, the use of intra-alveolar irrigation and curettage without complementary treatments showed poor results for pain control (8,27). These results may indicate that intra-alveolar irrigation procedures are required, but not sufficient to obtain an accepTable decrease in pain.

Among the treatments which fulfilled the pain reduction proposed parameters, the most frequent procedure was an intra-alveolar placement of therapeutic products, excepting the use of low-level lasers or magnetotherapy. One of them, Alveogyl® (Septodont, Cambridge, Canada) is a therapeutic paste that contains iodoform (antiseptic), butamben (anesthetic), and eugenol (analgesic). Eugenol generates pain reduction through an inhibition mechanism of glutamatergic neurotransmission, activation of tumor necrosis factor-alpha (TNFα), and the endogenous opioid system (28). Neocones® (Septodont, Saint-Maur-des-Fossés, France), is a dental Tablet that consists of polymyxin B sulfate (bactericidal on gram-negative), tyrothricin (bactericidal on gram-negative and spirochetes), and neomycin sulfate (broad-spectrum antibiotic). Nevertheless, the pain reduction is mainly linked to a local anesthetic compound of tetracaine hydrochloride (25). SaliCept Patch® (Carrington, Irving, USA), is a lyophilized product that contains an amorphous acemannan hydrogel (aloe vera plant filtrate). Acemannan gel is involved in macrophage activation, which stimulates fibroblast cytokine secretion and alveolar angiogenesis. TNFα and 1-interleukin are cytokines associated with anti-inflammatory effects and wound healing properties (29).

The aforementioned products (Alveogyl®, Neocones®, and Salicept Patch®) were specifically developed for the treatment of AO. Nonetheless, PRF has been successfully developed for other therapeutic applications (30) and then used for AO treatment. PRF is obtained from a sample of patients' blood drawn at the moment of AO intervention. It does not require anticoagulants or platelet activators. PRF could control pain through a biologic mechanism linked to leukocyte functions and alveolar growth factors secretion such as transforming growth factor (TGF), 1-interleukin, fibroblast growth factor (FGF), platelet-derived growth factor (PDGF), and TNFα. They increase and promote fibroblastic and angiogenic activity. Furthermore, the anti-nociceptive effects could be explained by the release of other substances like interleukins (4,10,13), opioid peptides (endorphin beta, metencephalin, dynorphins), and insulin-like growth factor type 1 (IGF-1), which plays a fundamental role in cell growth (31).

Low-level laser therapy (LLLT) was a highly effective treatment that did not need to place an intra-alveolar therapeutic product. The wavelength of these lasers for dentistry use ranges from 635 to 950 nm (32). The mechanisms for LLLT-mediated pain relief are not fully understood. Several possible mechanisms are believed to explain the effects of LLLT, such as increased production of endogenous opioid neurotransmitters and local blood circulation by accelerating cellular redox reaction. An increased threshold for thermal pain as well as an increase in the production of adenosine triphosphate and anti-inflammatory cytokines (33). The advantages (25,31,34-36) and disadvantages (35-37) of each treatment are summarized in Table 4.

Only one of the analyzed treatments used antibiotic medication for AO management. It is widely accepted that systemic antibiotics do not have a greater advantage than local measures in immunocompetent patients (5,38). On the other hand, considering that AO is a painful entity, analgesic-anti-inflammatory medication is a useful measure. Nevertheless, its use should not be considered more relevant than intra-alveolar procedures. The treatment developed by Chakravarthi (9), without overlooking that this was a non-randomized study with a critical risk of bias, showed one of the best pain control performances of the treatments included in this review. In the aforementioned study, AO patients were treated with an intra-alveolar placement of PRF and medicated with aceclofenac.

**Table 4:**
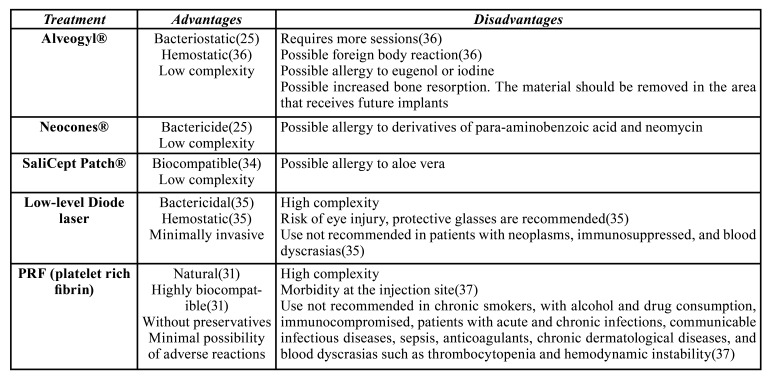
Advantages and disadvantages of alveolar osteitis treatments.

Thus, it could be determined that the use of PRF and aceclofenac showed a combined-synergic effect in pain reduction. Despite this, there are studies where aceclofenac was not effective in the control of postoperative pain (39,40), suggesting that pain reduction could be mainly due to intra-alveolar placement of PRF.

- Feasibility of clinical application

All AO treatments analyzed in this systematic review achieved pain reduction, although, with different time intervals, this is why all the treatments could potentially be applicable. The feasibility of clinical application of the best performance treatments should be considered according to their advantages and disadvantages (Table 4), operator training, equipment required for application, and cost-effectiveness analysis, among other variables.

- Limitations of the present review

This study focuses on pain remission in AO treatment. However, other important variables such as epithelial healing, bone exposition, and analysis of adverse effects, were not considered.

Further RCTs are needed in order to validate the best performance AO treatments analyzed in this systematic review. These studies should also consider other features such as a combination of intra-alveolar procedures, pharmacological schemes, risk factors for AO, and the record of clinical improvement variables (epithelial healing, bone exposition, necrotic debris, etc). Pain is a subjective experience, which means that it cannot be directly observed by those who are not experiencing it. This subjectivity generates a bias that is difficult to correct, since it is mainly due to the past experiences of individuals. This bias due to subjectivity conditions the quality of the trials. In future studies, emphasis should be placed on reducing the impact of subjectivity by controlling the pre-intervention and measurement of outcomes domains.

Conclusion

AO treatment could be categorized into basic (intra-alveolar irrigation) and specific procedures. The first ones should always be applied, and the second ones allow pain control success. There are invasive or non-invasive specific procedures, low or high complexity for the management of AO. The availability and the advantages or disadvantages of each could influence the selection of the therapeutic option.
